# Supplementary figures and images for: Formyl Peptide Receptor 2 Alleviates Hepatic Fibrosis in Liver Cirrhosis by Vascular Remodeling
Source: Int J Mol Sci. 2021 Feb 20;22(4):2107. doi: 10.3390/ijms22042107 (PMC7924385; doi:10.3390/ijms22042107)

Supplement Fig. 1

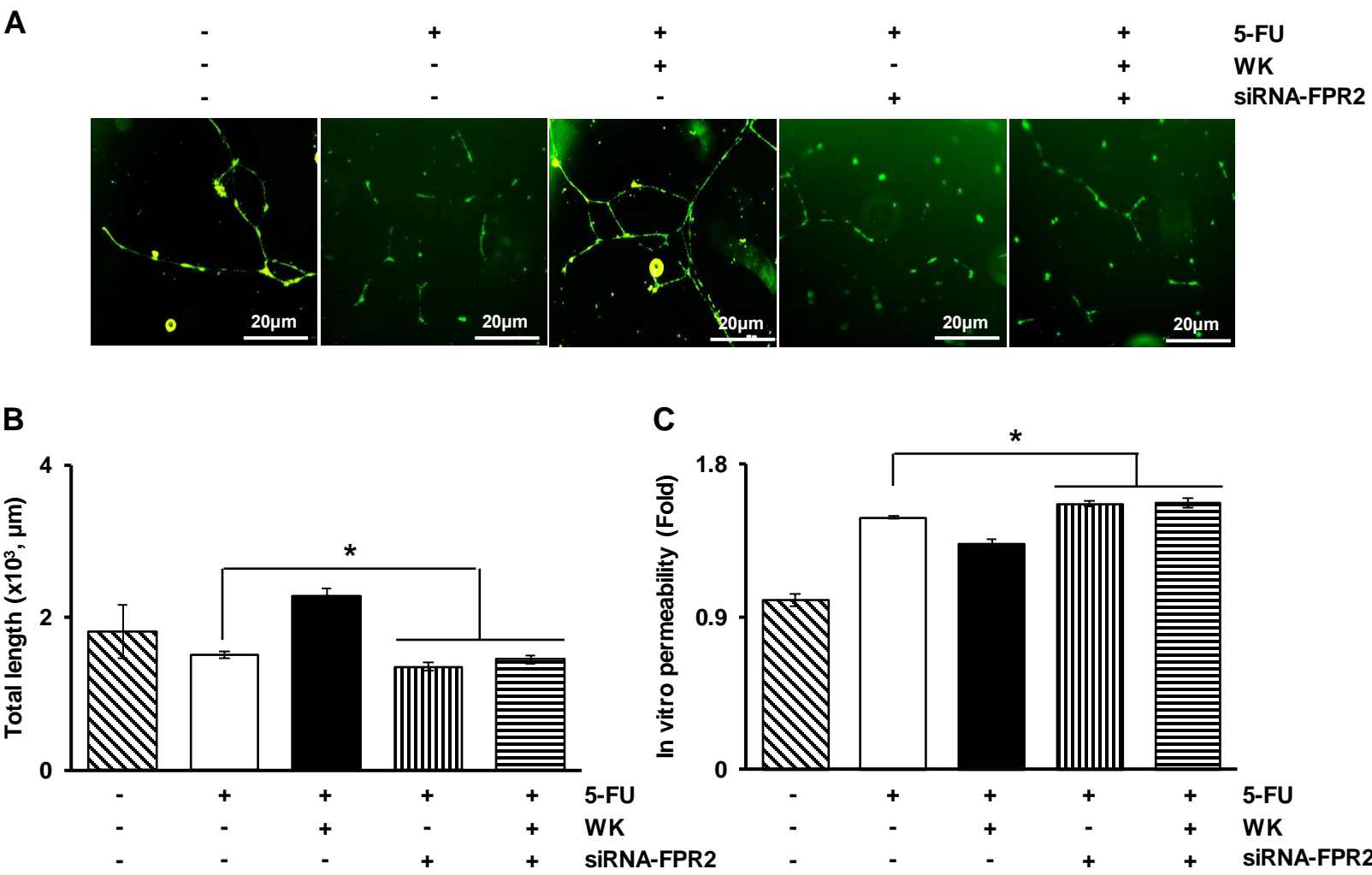

Supplement Fig. 2

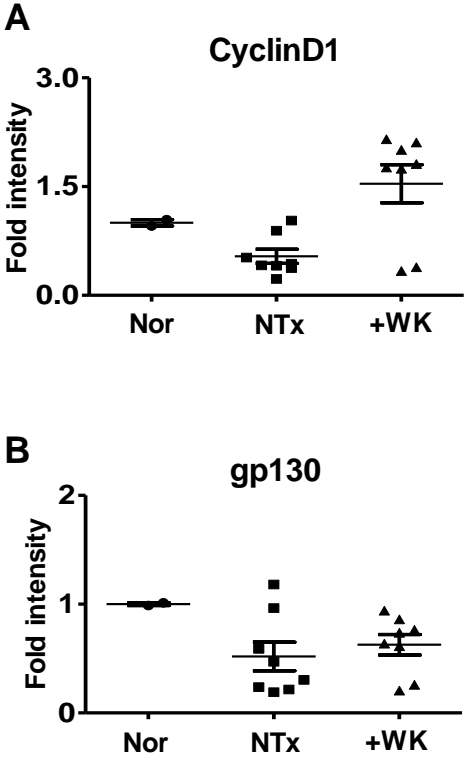

Supplement: Supplementary file 1 [file ijms-22-02107-s001.pdf]
